# Atonal Music: Can Uncertainty Lead to Pleasure?

**DOI:** 10.3389/fnins.2018.00979

**Published:** 2019-01-08

**Authors:** Iris Mencke, Diana Omigie, Melanie Wald-Fuhrmann, Elvira Brattico

**Affiliations:** ^1^Department of Music, Max Planck Institute for Empirical Aesthetics, Frankfurt, Germany; ^2^Center for Music in the Brain, Department of Clinical Medicine, Aarhus University and The Royal Academy of Music, Aarhus, Denmark; ^3^Department of Psychology, Goldsmiths, University of London, London, United Kingdom

**Keywords:** atonal music, pleasure, uncertainty, predictive coding, aesthetic experience

## Abstract

In recent years, the field of neuroaesthetics has gained considerable attention with music being a favored object of study. The majority of studies concerning music have, however, focused on the experience of Western tonal music (TM), which is characterized by tonal hierarchical organization, a high degree of consonance, and a tendency to provide the listener with a tonic as a reference point during the listening experience. We argue that a narrow focus on Western TM may have led to a one-sided view regarding the qualities of the aesthetic experience of music since Western art music from the 20th and 21st century like atonal music (AM) – while lacking a tonal hierarchical structure, and while being highly dissonant and hard to predict – is nevertheless enjoyed by a group of avid listeners. We propose a research focus that investigates, in particular, the experience of AM as a novel and compelling way with which to enhance our understanding of both the aesthetic appreciation of music and the role of predictive models in the context of musical pleasure. We use music theoretical analysis and music information retrieval methods to demonstrate how AM presents the listener with a highly uncertain auditory environment. Specifically, an analysis of a corpus of 100 musical segments is used to illustrate how tonal classical music and AM differ quantitatively in terms of both key and pulse clarity values. We then examine person related, extrinsic and intrinsic factors, that point to potential mechanisms underlying the appreciation and pleasure derived from AM. We argue that personality traits like “openness to experience,” the framing of AM as art, and the mere exposure effect are key components of such mechanisms. We further argue that neural correlates of uncertainty estimation could represent a central mechanism for engaging with AM and that such contexts engender a comparatively weak predictive model in the listener. Finally we argue that in such uncertain contexts, correct predictions may be more subjectively rewarding than prediction errors since they signal to the individual that their predictive model is improving.

## Introduction

Music listening has the potential to evoke strong and intense experiences in listeners, and music-making is an activity that is present across all cultures. Music is, however, highly diverse such that the treatment of its features, like the spatial and temporal organization of pitches (i.e., tonality and rhythm), timbre, and form, diverge dramatically not only across but also within any given culture and epoch. Despite this immense heterogeneity, however, an ever-increasing number of scientists feel compelled to investigate the cognitive, affective, and neural correlates of music listening (Juslin and Sloboda, 2010; Koelsch, 2012).

While this research is still in its infancy, two trends can be identified and serve as a motivation for the current thesis: namely that investigating specifically Western art music from the 20th and 21st century^1^ can make a key contribution to the field. The first trend is a general tendency for music neuroscience and music psychology (Levitin and Tirovolas, 2009) to use frameworks that either emphasize the role of emotions in music processing (Koelsch et al., 2006; Juslin and Sloboda, 2010) or that take a more cognitive perspective with a focus on similarities between music and language (Patel, 2003; Levitin and Tirovolas, 2009). In other words, there is an observable tendency for previous work to avoid engaging with music as an aesthetic object. As previous dominant frameworks are dedicated to investigating basic brain principles, they rarely seek to illuminate the hedonic and evaluative processes that are unique to a genuinely aesthetic experience of music, an established concept in the field of neuroaesthetics and music psychology (Juslin and Sloboda, 2010; Nusbaum and Silvia, 2011; Brattico et al., 2013; Brattico P. et al., 2017; Jacobsen and Beudt, 2017). In a nutshell, by applying ‘aesthetic’ to the music listening experience, we seek to emphasize the accompanying mechanisms and factors that are specific to an art context.

A second observable trend is the tendency for existing psychological models of aesthetic experience of music (Konečni, 2005; Hargreaves, 2012; Juslin, 2013) to concentrate on music of the Western tonal system (Eerola, 2012; Koelsch, 2012; Janata, 2013; Zivic et al., 2013; Bharucha, 2014) thereby capturing only a very limited scope of musical genres and phenomena. As neuroscience research has mainly used stimuli from instrumental classical music, recent proposals by Brattico and colleagues point to the necessity of including in the discussion a greater variety of genres and styles in order to provide a more comprehensive account of the musical aesthetic experience (Brattico et al., 2013).

Taken together then, notably excluded from current empirical efforts and theoretical accounts are considerations of both music from non-Western cultures and the musical styles that are addressed in this paper as aesthetic objects requiring comprehensive and systematic investigation. Thus, while an increasing number of studies have begun to clearly acknowledge that music is heterogeneous and that an enormous variety of effects may emerge from the diversity of musical genres and styles (Krumhansl et al., 2000; Brattico et al., 2011; Salimpoor et al., 2011; Alluri et al., 2012, 2015, 2017; Zatorre and Salimpoor, 2013; Dean and Bailes, 2016; Dean and Pearce, 2016; Poikonen et al., 2016), there is still a need to stress the importance of an approach that treats music purely as art, by encompassing arguably the most *innovative*, *experimental* and *challenging* of musical styles, namely 20th/21st century art music.

Exactly for the reasons stated above we propose that 20th/21st century art music represents a promising topic for future research into music listening as an aesthetic experience^2^. Through atonal music (AM), the set of styles or musical “languages” that have been investigated until now can be expanded on. Research on AM has the potential to reveal neural mechanisms that might be unique to the aesthetic experience of music, as opposed to those focused on rule-based processing of structure in Western tonal music (TM). Given that it is, at first glance, a potentially unpleasant environment, we argue that AM offers one especially promising tool with which to discover how experiences that might be avoided in everyday situations can be appreciated in an aesthetic context: a mystery that leads right into the notion of the transformative power of art which is so prominent in modern Western thinking.

In the following sections, we first historically contextualize 20th/21st century art music, before reviewing the psychological research that thus far directly or indirectly addresses AM. The subsequent section describes, by means of music theoretical analyses and music information retrieval methods, how a specific kind of AM (that we expand on in the next sections) differs from Western TM. We then examine a variety of factors and mechanisms that may influence the enjoyment of AM. We group these into three categories namely person related factors, extrinsically driven factors and intrinsically driven factors. The terms extrinsic and intrinsic are used to denote the origin of information that influences the kind of pleasure derived from the music. Specifically, with *extrinsic factors* we refer to mechanisms that rely on information external to the stimulus. In contrast, mechanisms subsumed under *intrinsic factors* rely on information processing that is stimulus internal. Following a synthesis of mechanisms we conclude with future prospects regarding the role we suggest 20th/21st century art music should play in future research in the field of neuroscience of music.

## Western Art Music From the 20th And 21st Century

### A Brief History

The origins of 20th/21st century art music can be dated as far back as the late 19th century when compositional innovations were characterized by a gradual dissolution of the Western tonal system, which in turn had been based mainly on the major-minor tonality developed in the 17th century (Dahlhaus, 1967, 1990). However, the clearest steps toward consolidating the field of 20th/21st century art music were made by Arnold Schoenberg (1874–1952), who gradually moved away from using tonality and traditional keys in his compositional works and in finally proclaiming the “emancipation of dissonance” (Dahlhaus, 1978). He may be considered one of the leading figures of the avant-garde of the 20th century. While in the early phase, Schoenberg’s compositions followed so called “free atonality,” he later invented the more structured twelve-tone technique around 1920, which aimed at treating all twelve tones within an octave as equivalent in a musical piece (Dibelius, 1988). This resulted in music that was strongly dissonant and which lacked a tonal hierarchical structure – a characteristic that composers aimed for. An even sterner approach was then developed by the serialists of the 1950s (central figures being O. Messiaen, P. Boulez, K. Stockhausen, L. Nono - also protagonists of the Darmstadt School), who not only pre-determined the use of pitches as Schoenberg did, but also transferred this method of equal treatment to other parameters like dynamics and duration of tones, resulting in a strict “parametric thinking” (Grant, 2001, p. 62).

While Schoenberg and the Serialists founded compositional “schools” in the first half of the 20th century, the latter half witnessed an ever increasing individuality and pluralism of musical styles and forms (Hüppe, 2016). Furthermore, compositional innovation emerged on several levels other than melody and harmony, including rhythm, timbre, and form. With regard to innovations in form, for instance, very short pieces like Stockhausen’s early piano pieces (*Piano Piece III.* lasts 40 s) were composed alongside pieces like *Organ2/ASLSP* by John Cage (composed to last a few centuries). With regard to innovations in timbre, the musicality of noises (*musique concrète*) was explored alongside the noisiness of classical instruments. By inventing *musique concréte instrumentale*, Helmut Lachenmann (^∗^1935) radically re-thought how instruments could be played (e.g., using the back of the bow on string instruments). The use of electronic means to produce tones expanded this field even more (e.g., Stockhausen in the 1960s).

These examples of powerful artistic innovations and the occurrence of new aesthetic paradigms in each decade are typical of Western art music from the 20th and 21st century. This innovative strength can be seen as its teleological drive which was most influentially formulated by Th. W. Adorno in his *Philosophy of New Music* (Adorno, 1949). This drive originated from the aesthetic thinking of Schoenberg who claimed that music composition must overcome academic norms and old categories and that the composer similarly must be strongly critical not only toward the previous but also toward the popular and the masses (Utz, 2016). While these transformative processes are typical for paradigm shifts, in the context of ‘New Music’ (as termed by Adorno), it became an ongoing aesthetic premise (Hüppe, 2016). Creating music that overcomes old listening habits by means of presenting something entirely new was a central motivation for artists. However, for the listener this meant being presented with a vast diversity of musical vocabulary that represents an enormous challenge for the listening process.

### Contemporary Classical Music – An Artistic Niche?

Nowadays, in keeping with the above-described aesthetic premise, a great deal of instrumental contemporary classical music is based on the free treatment of tonality, meter and form. However, while composers utilize independent and often self-designed systems in order to create regularity as a base for their compositions, this does not always facilitate the listening process. An additional challenge faced by the listener is identifying clear musical genres, which would serve as a stylistic reference point and which would provide information for pre-classification (Leder et al., 2004). Certainly, the demanding nature of this music has contributed to the status of contemporary classical music as a niche in modern cultural life (Ball, 2011). However, the last 30 years have seen a huge growth in the number of music ensembles specializing in the music of the 20th and 21st century (Fricke, 2011). Even though this musical style does not attract audiences as large as pop or classical music concerts, systematically including music from the 20th and 21st century is high on the agenda of most large concert houses where “hybrid concepts,” i.e., programs consisting of both classical and contemporary classical music (Maletz, 2011, Chapter 2), often receive significant funding (Fricke, 2011, p. 174). Moreover, there are several ensembles in Europe which focus solely on 20th/21st century art music, like the Ensemble Modern (Germany), Ensemble Intercontemporain (France), Asko/Schönberg (Netherlands), Klangforum Wien (Austria), and London Sinfonietta (United Kingdom), to name but a few. Therefore, although this style of music appears to be a niche and is less popular than other musical styles, it represents an impressively rich and highly diverse musical phenomenon (Hüppe, 2016). Contemporary classical music nowadays is a highly interactive artistic area frequently linked to other art sectors, like fine arts, installation and performance art (Bishop, 2005). Thus, contemporary classical music can be seen as one of the driving forces of the avant-garde and contemporary art creation.

### Aesthetic Values of 20th/21st Century Art Music

As indicated in the previous sections, creating and presenting something truly novel plays a crucial role in most 20th/21st century art music. This musical newness can partially be seen as a critical factor for the mere listening experience but also for the appreciation of it. Similarly, this music’s aim, namely to sensitize the listeners’ hearing/audition and to literally overcome perceptual habits, can inform our understanding of the positive aesthetic values, the underlying appreciation and the “sensory attractiveness” (Utz, 2016) that accompanies a positive aesthetic experience of AM.

In the context of journalistic writings about contemporary classical music, the music is often described as a music type that presents something new. Moreover, pieces from the 20th/21st century often require excellent and highly developed playing skills, which can induce fascination and therefore a positive valuation by the audience^3^. Finally, the musicians’ motivation for playing contemporary classical music is linked to free experimentation with and exploration of new sounds, the close collaboration with composers and importantly the possibility to present something that has never been heard before (Meißner and Morawek, 2012). Curiosity, energy, and openness (Meißner and Morawek, 2012) are often reported as being necessary for playing – and also enjoying – contemporary classical music. Thus, novelty, innovation, and the challenge it affords may all be conceptualized as positively valued and may guide the appreciation of contemporary classical music.

## State of Research – Atonal Music in the Empirical Music Literature

Decades of psychological research have shown that a tonal musical context has a strong psychological representation, whereas musical events that do not occur in a tonal context are less stably represented (Krumhansl, 1979). For instance, while listeners are able to pick correct reductions of tonal excerpts in tonal contexts, they are not able to do so with atonal excerpts (Dibben, 1994). Atonal music does not provide a tonic reference point. Nor does it provide corresponding chords of significant but lower importance. This lack of pitch hierarchy not only has perceptual but also cognitive implications. Indeed, Schulze et al. (2012), when testing memory for tonal and atonal melodies with a paradigm in which the sound sequences had to be kept in memory, found that both musicians and non-musicians performed significantly better in the tonal sequences. The authors reasoned that structured material present in the tonal task enables better working memory performance (Lerdahl and Jackendoff, 1983). In adults, it has been shown that it is more difficult to recognize transposition in the context of novel atonal relative to tonal melodies (Cuddy et al., 1981; Dowling et al., 1995).

The notion that the brain tracks hierarchical structures (e.g., Nelson et al., 2017) and that such hierarchical structures facilitate the storing and recalling of information has been evidenced in many different domains (e.g., Mandler, 1984). Specifically, in the domain of music processing it has been shown that the tonic, the subdominant and the dominant strongly influence processing speed (Tillmann et al., 2008). These three chords of music are known as the “harmonic core” and represent the pillars of tonal hierarchical organization in Western TM from the 17th century onward (Dahlhaus, 1967; Bharucha and Krumhansl, 1983). This hierarchy is inherently linked to the tonal scale, in which every tone within an octave has a specific function. The first tone of the scale, called the tonic is the “head of the hierarchy” (Krumhansl and Cuddy, 2010) and represents the auditory and cognitive reference point (Rosch, 1975). All other functions follow this reference in a hierarchical manner (within one key). Even though this “pitch centrality” can be found across musical styles and cultures (Krumhansl and Cuddy, 2010), AM might represent an exceptional case due to its equal treatment of all tones within an octave and the resulting lack of a clear tonal center.

An interesting insight into how the brain deals with atonal excerpts may be drawn from examining the strategy listeners use to remember the excerpts (Mikumo, 1992). In one task, musically trained subjects were required to compare melodies that had been interrupted by different “retention intervals” and to report on their encoding strategies. Results revealed that for tonal melodies, participants used a verbal (e.g., note naming) strategy whereas for the atonal contexts they used “rehearsal strategies (such as humming and whistling)” (Mikumo, 1992). In addition to storage, another interesting insight may come from how atonal sequences are processed online in comparison with tonal ones. It has been shown, through research on auditory scene analysis (Bregman, 1990) and grouping mechanisms in music perception (Deutsch, 1999), that grouping in music facilitates recognition, processing and recall of melodies or chunks of items (Creel et al., 2004). Importantly, such structures are held to be key to the formation of predictions (van de Cruys and Wagemans, 2011).

There is only very little research on how predictions are generated in atonal musical contexts, but it has been suggested that listeners generate expectations in a contrary manner. Specifically, Ockelford and Sergeant (2012) showed – based on probe-tone experiments with atonal phrases – that listeners expect the “opposite” to happen, in other words applying an “antistructure” toward future musical events. In Ockelford’s experiments (2012), it was found that after listening to a 12-tone series, recipients expected that none of the already-heard sounds would be repeated, and that none of the already-heard sounds would suggest the existence of a key. These findings are in line with those from Krumhansl et al. (1987), suggesting that listeners use tonal schemas when listening to AM. Generally, in comparison to tonal major/minor contexts, atonal contexts evoke weaker expectancies (Vuvan et al., 2014) although the organization of AM seems to be recognizable by listeners (Clarke and Krumhansl, 2017), and particularly the segmentation of sound is based on similarity and/or contrast (Deliège, 1989). Research from cross-cultural studies suggest that familiarity and enculturation with a particular musical style shapes expectancy processes (Curtis and Bharucha, 2009; for a review on cross-cultural music perception and cognition see: Stevens, 2012). This suggests that familiarity with AM might equally modulate expectancy mechanisms in the listening process.

With regard to how the high degree of dissonance (simultaneous and subsequent) in AM is received, research on processing of and preference for consonance vs. dissonance cannot be ignored, particularly when considering prediction mechanisms in music. Research suggests that the preference for consonance chords stems from its harmonicity. Consonant dyads have many frequency components in common (Dewitt and Crowder, 1987; Tramo et al., 2006; Ebeling, 2008). Moreover, their partials contain smaller integer ratios than those of dissonant dyads. In dissonant intervals, the harmonics are dissimilar and the frequency components lie in the critical bandwidth of the cochlea (Greenwood, 1991). For the listener, this leads to the perception of auditory roughness (Plomp and Levelt, 1965) which underlies sensory unpleasantness. Another hint at the preference for simpler frequency ratios is the finding that most of the scales used for making music consist of 5–7 tone scales which correspond to the harmonic series (Gill and Purves, 2009). However, in contrast to what is suggested by the dichotomy ‘consonance vs. dissonance,’ no clear line can be drawn between them as there is an acoustical as well as perceptual continuum between the simplest and most complicated frequency ratios.

Preference for more consonant sounds nevertheless seems to start at very early developmental stages with newborns opting to listen to consonant rather than dissonant intervals (Zentner and Kagan, 1998; Trainor et al., 2002) and a link has been made between the less robust neural responses to dissonant relative to consonant chords that are found at the brainstem level (Bidelman and Krishnan, 2009). To which extent and in which way these preferences are shaped by learning and culture is a long-debated issue, a detailed examination of which is outside the scope of this paper. However, we discuss the role of learning in the context of mere exposure effects in following sections.

While behavioral research on perceptual and cognitive processing of atonal melodies has been minimal, even less research on AM has been carried out in the neuroscientific domain. Indeed, very striking is that when AM is used in such studies, it serves only rarely as a style of music in its own right but as examples of unpleasant dissonant music (Blood et al., 1999; Gagnon and Peretz, 2000; Flores-Gutiérrez et al., 2007), as a less preferred musical genre (Cross et al., 1983; Smith and Melara, 1990), as stimuli to induce “‘fearsome’ emotions” (Flores-Gutiérrez et al., 2007) or as a control stimulus for “non-musical auditory inputs” (Trost et al., 2012, p. 2770). However, a few studies have indeed examined AM as a musical style in its own right. One particularly relevant approach has taken an information theoretical perspective to atonal and serial music and has shown the entropy of such melodic excerpts to be high and the information content of individual events to be low (Dean and Pearce, 2016). Indeed, due to the lack of a tonal center constituting an auditory reference point, individuals listening to AM may be expected to experience high “predictive uncertainty” (Hansen and Pearce, 2014). Similar characterizations can be traced back to the music philosopher Leonhard Meyer, the first to apply information theoretic notions to theories in music cognition when he proposed that a musical style is cognitively represented as a network of probabilities in the listener’s brain (“internalized probability systems,” Meyer, 1956, p. 414). An information theoretical perspective (Pearce, 2005; Huron, 2006) will later serve as a foundation to lay down our hypotheses regarding how pleasure might be derived from an encounter with AM.

## How Is Atonal Music Different? Single Piece and Corpus Analysis

The following part of this paper moves on to compare tonal with AM from different methodological angles, in order to illustrate concretely the potentials of AM. While we use music theoretical analyses to give a functional analysis as well as to provide a visual illustration of the scores of the musical excerpts, the analyses conducted with the MIR (music information retrieval) toolbox for Matlab (Lartillot and Toiviainen, 2007b) have the purpose to calculate particular features both in single pieces and in a corpus analysis. These results shall demonstrate characteristics of AM from a computational perspective. The focus here is on tonality and meter rather than on timbre and form as these features represent the most salient characteristics of a musical piece in terms of its texture. While *tonality* refers to the vertical and horizontal relationships between the pitches (i.e., melody and harmony), *meter* relates to the temporal organization of the pitches. In contrast, *timbre* represents the specific character and quality of sounds, while *form* refers more to the overall structure of a musical piece.

### Single Piece Analysis by Means of Music Theory

In order to represent AM, we chose pieces that do not follow the rules of traditional harmony and therefore lack a clear tonal center as well as a metrical regularity. However, to not restrict the selection to a single musical epoch, we consciously chose pieces that belong to different sub periods of 20th/21st century music (see Table 1). For the Western tonal styles, we selected pieces from the so-called common practice period (encompassing the baroque, classic and romantic epoch but often subsumed simply under the term classical music: 1600–1910; Hyer, 2001), showing a clear tonal center and an equally strong metric regularity. Table 1 shows the selected pieces including composer, date of composition, corresponding sub-period and period.

**Table 1:** Overview of selection of pieces and periods.

Composer	Piece	Date of composition	Subperiod	Period	MIR excerpt in seconds
J. S. Bach	Goldberg Variations, Variatio 12	1741	Baroque	Classical music	0–30
W. A. Mozart	Piano Sonata No. 8, K. 310	1778	Classic	Classical music	0–30
F. Chopin	Waltz, A-Minor	1843	Romantic	Classical music	0–30
K. Stockhausen	Piano Piece No. VIII	1954	Serialism	20th/21st century music	0–30
W. Rihm	Piano Piece No. 5	1975	New Simplicity	20th/21st century music	0–30
J. Widmann	Fleurs du Mal	1996/97	Contemporary	20th/21st century music	49–79


*The selection of pieces had two aims: (1) Classical pieces had to be strongly tonal and highly regular in meter. Pieces from the 20th/21st century had to be highly atonal and similarly highly unregular in meter (we refer to them as “atonal pieces”). (2) We then aimed at covering a scope of musical styles as broad as possible. Therefore we took one piece of each of the main subperiods from the classical (1700–1850) and the 20th/21st century period (1910–2000). Moreover, it is indicated which parts of the pieces were chosen for analysis: “MIR excerpts in seconds” refers to the excerpt we chose for automatic feature extraction by MIR toolbox for Matlab (Lartillot and Toiviainen, 2007a; see Figure 2). For this we took the first 30 s of the audio recordings, except for the Widmann piece, as it starts with a 50-s long repetition of a single note (accordingly the score excerpt of the Widmann piece displays exactly this part).*

**FIGURE 1:**
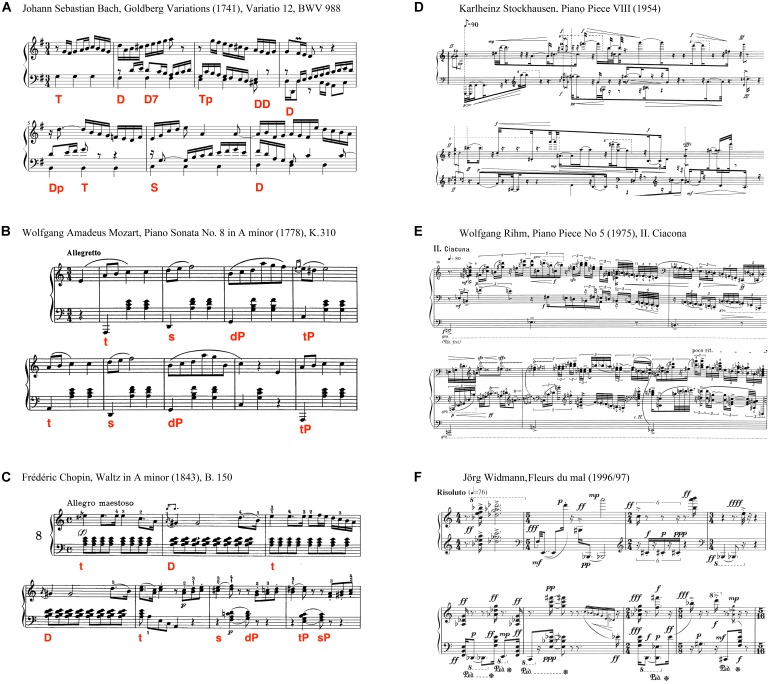
Single piece analysis by means of music theory. Metrical regularity in classical pieces is readable in the strictly kept time signature at the beginning, the equal lengths of tones and the repetitive accompaniment in **(B,C)**. In contrast, atonal pieces **(D–F)** show different time signatures and a large variety of note lengths, resulting in a complex rhythm without a regular meter. Tonality in the classical pieces is indicated by the set-up of the home key in the beginning. In **(A)** the key of *G major* is prevalent in the first bar, being presented in a scale, supported by the G in the bassline. In the next bar the key switches to the dominant, *D major*, supported by the third (*F#*) in the bassline. In **(B,C)**, which reveals a homophonic texture (in contrast to a polyphonic texture in **A**), the clear key structure is conveyed mainly by the chordal accompaniment, where each chord belongs to a particular function within the home key. The piece in **(C)** starts with the tonic (‘t’, *A minor*, 1st bar), followed by the subdominant (‘s’, *D minor*, 2nd bar), followed by the parallel of the dominant (dP) *G major* with a seventh, which leads to *C major*, the major parallel key of *A minor* (‘tP’). This tonal structure is then repeated. In contrast, the atonal pieces reveal a completely different texture, leading to a more flexible structure that is first of all lacking triad-building chords. However, the chords that appear are highly dissonant. This becomes clear with looking at intervals. The first interval of **(D)** displays a *G#*, *A* and a *F#* in parallel leading to a highly dissonant sound especially due to the second. The third sound of **(E)** consists of six notes in parallel, similarly dissonant, again partly due to a minor second between *E* and *F*. Moreover, also consequent intervals suggest a dissonant, thus atonal structure. For instance in **(F)** the second bar consists of a ninth, 2 minor sevenths, 1 major seventh and a tritone, thus covering most dissonant intervals.

Beginning with meter, the scores (Figure 1) show that the metrical regularity in the classical pieces (Figures 1A–C) stems first and foremost from the strictly followed time signature. By contrast, most of the atonal pieces (Figures 1D–F) lack a regular pulse. Moreover, in Widmann’s *Fleurs du mal*, the time signature changes in each bar which results in an irregular stress pattern. Another sign of the irregular meter in the atonal pieces are the highly diverse note lengths. For example, in Stockhausen’s piano piece, a wide range of note lengths appears, beginning from a half-note as the first note until sixty-fourth notes with additional triplets and sextuplets. In contrast, the classical pieces use less diverse note lengths, as in the piece by Bach, where mainly sixteenth notes run over a fundament of crotchets. Further, in the pieces by Mozart and Chopin, the metrical regularity derives heavily from the bass accompaniment with its constant pulse of quavers (Mozart) or crotchets (Chopin). Finally, while in the classical pieces the lower and upper voice share the same meter, in the atonal ones, best seen in the Stockhausen piece, both voices are treated completely separately.

With regard to tonality, the scores demonstrate key differences between the classical and atonal pieces. In the classical pieces, the home key is set up right in the first bar. Moreover, a different function within the key appears within the following bars (for a functional analysis see Figure 1). This cadence structure is one of the most important features of classical and Western tonalities and is closely correlated with the structural unfolding of the melodic subjects and the piece as a whole. Since the cadence is based on sequences of triads and tetrads, those pieces are additionally characterized by a high degree of sensory consonance. In contrast, the metrically less structured atonal pieces are accompanied by a different treatment of tonality whereby cadence-like chords are lacking. Indeed, rather than having a symmetric intervallic structure, a highly diverse range of dissonant intervals occur, leading to a high degree of intervallic entropy (for a more detailed analysis see Figure 1, caption).

Finally, a very striking difference between the classical and atonal pieces is their Gestalt quality, again observable from the score. The main Gestalt laws are proximity and similarity (van de Cruys and Wagemans, 2011), and Gestalt theory plays a key role in the current understanding of music perception (Bregman, 1990; Reybrouck, 1997; Deutsch, 2013). Whereas the classical pieces provide ascending and descending scales and thus avoid large jumps between notes, the atonal pieces, in particular Rihm’s piano piece (Figure 1D), are characterized by large jumps and frequent changes of melodic direction thus impeding easy groupings based on Gestalt principles^4^. Moreover, both with regard to the note lengths mentioned previously and the melody lines, it is very clear that the classical pieces tend to show a high self-similarity in comparison with the atonal pieces (Narmour, 1990). This is in line with results of a corpus analysis which showed evidence that classical music is characterized by small pitch tendencies (Huron, 2001).

### Single Piece Analysis by Means of Automated Extraction of Features

For the purpose of describing music with quantitative and automated methods we used the MIR toolbox for Matlab (Lartillot and Toiviainen, 2007b), which has been used in recent neuroscientific studies on music (Brattico et al., 2011; Brattico P. et al., 2017; Alluri et al., 2012, 2017; Bogert et al., 2016; Burunat et al., 2016; Poikonen et al., 2016). The tool allows the tracking of continuous developments of changes in both low-level features, including spectral composition, roughness and timbre, as well as high-level features, such as tempo, pulse clarity and key clarity values. The MIR approach is useful for the investigation of the relationship between perception, brain responses and musical structure since the dynamic nature of musical stimuli requires a clear quantification of features over time. We chose two features from the MIR toolbox to describe tonality and metrical regularity of musical pieces, using the functions key clarity and pulse clarity.

Pulse clarity function estimates the rhythmic clarity indicating the strength of the beats estimated based on interonset intervals. An autocorrelation function used in a constrained time-window assesses the self-similarity and thus gives the degree of pulse clarity. High pulse clarity indicates a regular and low pulse clarity an unregular metrical structure. The key clarity function is calculated based on pitch chromagram and the Krumhansl–Kessler algorithm of matching pitch class profiles to key profiles (Krumhansl, 1990; Toiviainen and Krumhansl, 2003). Both key and pulse clarity measures can provide a quantitative value of the perceptual experience by the listener. In fact, both have been shown to be highly correlated with perceptual ratings of individuals (Alluri et al., 2012). In the current analyses of the six pieces, we used a window size of 5 s and a hop factor of 33% for both key and pulse clarity (Recordings used see Supplementary Table 1).

**FIGURE 2:**
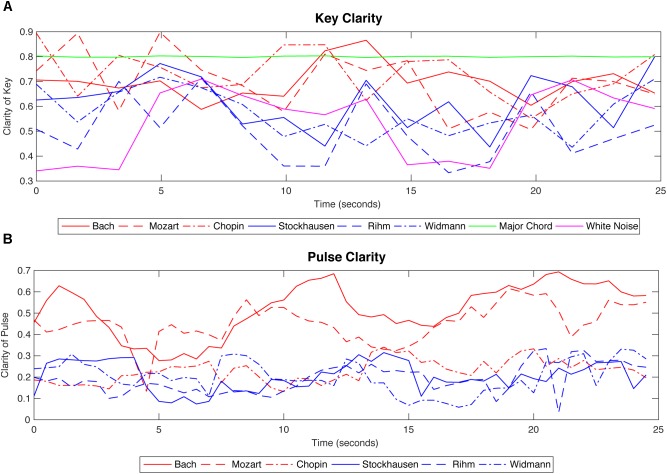
Single piece analysis of features (key and pulse clarity) by means of automated extraction. This figure displays the results of the analysis of single pieces with MIR Toolbox for Matlab (Lartillot and Toiviainen, 2007a). Red lines represent classical pieces, blue lines atonal pieces. In **(A)** results for key clarity are displayed, using a window size of 5 s and a hop factor of 33%. Even though at some points the values of modern pieces lie above classical piece values, classical pieces tend to obtain higher values over time (compare Table 2). In order to demonstrate the scope of key clarity that the algorithm is able to reveal we included a *C Major* chord (yellow) and white noise (green) into our analysis as reference points. Even though the representation particularly of the white noise indicating peaks and lows extracted from a constant signal can be seen as critical, it gives a reference of how to evaluate the key-related feature extraction of the pieces (see Table 2 for absolute and mean values of key clarity). In case of pulse clarity **(B)**, classical pieces obtain much higher values than atonal pieces and results are more significant. However, the Chopin piece shows lower values, which can be explained by the rubato interpretation of the musician, which generates slight changes in tempo at formal borders within a piece (e.g., “ritardandos” = deceleration). Particularly, at around 10 s, a drop of pulse clarity can be seen which is reflecting the deceleration of the pianist at the end of the first presentation of the main motif.

Key clarity shows how strongly a key is represented at any given moment. We would thus expect higher values for classical pieces due to their tonal constitution and lower values for the atonal pieces since they do not convey a key as clear as their tonal counterpart. Indeed, Figure 2A and Table 2 demonstrate this to be the case. However, it may be observed that the piece by Bach achieves a rather low key clarity value compared to the other two classical pieces. This can be explained by the fact that this contrapuntal piece consists of different, independent voices, intertwined with each other (as compared to homophony where one main voice is accompanied by a mainly chordal baseline, as is the case in Figures 1B,C). Due to the specific implementation of key clarity in the MIR Toolbox (see description above) a swiftly moving diatonic melody (in the given 5 s window) as can be found in the Bach piece will result in more saturated pitch class profiles and hence reduced key clarity. More specifically, the passing tones (on diatonic scale degrees 2, 4, 6, and 7) will be more emphasized compared to the triad tones (scale degrees 1, 3, 5), thus the clarity of the key profile decreases. The algorithm is not able to differentiate between low-density chromaticism and high-density diatonic movement of counterpoint. This can also be seen in the higher key clarity of the Stockhausen compared to the other atonal pieces due to its looser texture and lower event density. We included a *C Major* chord and white noise into our analysis in order to demonstrate the limits of this algorithm (see Figure 2A).

**Table 2:** Key clarity values.

Musical style		Mean key clarity	STD	Mean for musical style	Event density per second
	C-Major chord	0.7987	0.0034		

	J. S. Bach	0.6987	0.0709		3.778
Tonal Music	W. A. Mozart	0.6951	0.1217	0.7085	1.8388
	F. Chopin	0.7317	0.0972		1.5714

	K. Stockhausen	0.6206	0.1122		0.53494
Atonal Music	W. Rihm	0.5034	0.1278	0.5667	2.4072
	J. Widmann	0.5761	0.0937		0.83584

	White Noise	0.5048	0.1270		


*Tonal pieces on average obtain higher key clarity values than atonal pieces, as the “mean for musical style” suggests. However, in order to show limitations of the algorithm which the toolbox computes key clarity values with, we included a C-Major-chord and white noise into the analysis, expecting highest value representation in the case of the chord and lowest values for the white noise. Those scores represent actual frame of values one can expect from this algorithm.*

Pulse clarity provides estimations of the regularity of rhythmic or metrical pulsation (Lartillot et al., 2008). In Figure 2B the classical pieces achieve much higher values than the atonal pieces, whereas the piece by Bach obtains overall highest values, likely due to the regular movement in sixteenth notes (see Figure 1A). To explain the low scores of the Chopin piece, we assume that the specific rubato interpretation by M. Takedo-Herms is the reason. “Ritardandi” are a style-adequate feature in Romantic performance practice that consists in retardations of tempo played at formal borders, like phrase boundaries, which inevitably result in an attenuation of the clarity of the pulse.

### Corpus Analysis of 100 Piano Piece Excerpts

Here, due to our interest in the structural (and textural) differences between Western tonal and AM, we sought to compare tonality and meter across the styles. We accordingly selected types of music that we considered to represent exactly those two features clearly.

**FIGURE 3:**
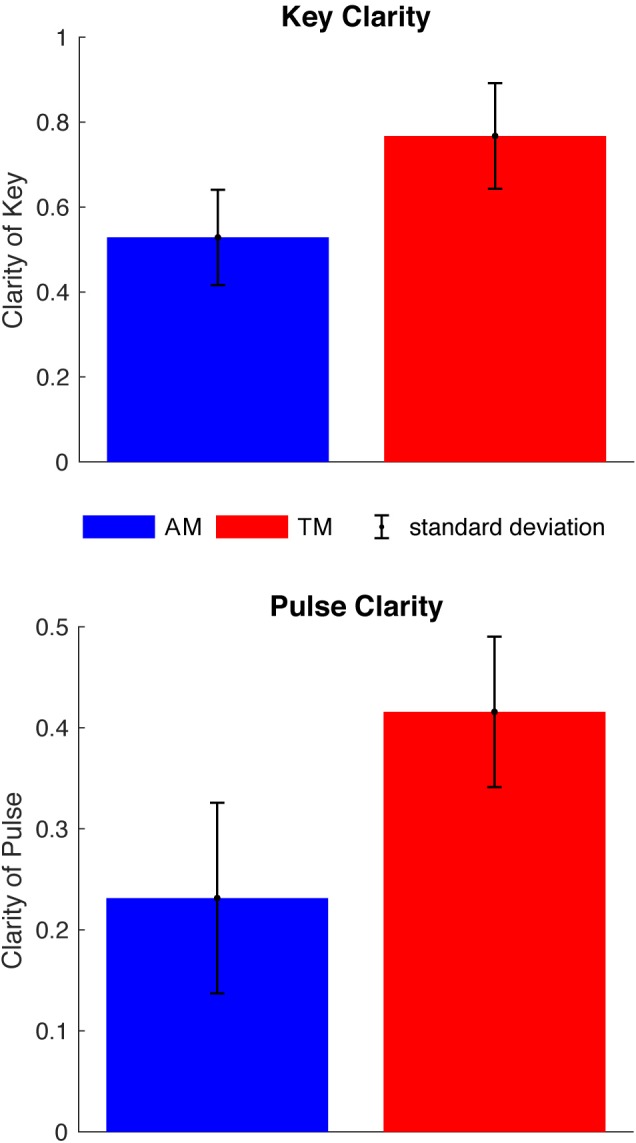
Corpus analysis of 100 piano pieces. Figure shows the mean key and pulse clarity values for each musical style, extracted with MIR Toolbox for Matlab (Lartillot and Toiviainen, 2007a). Two independent-samples *t*-tests were conducted to compare the two corpora (AM = atonal music; TM = tonal music). There was a significant difference in key clarity values for atonal (*M* = 0.5, *SD* = 0.1) and tonal (*M* = 0.8, *SD* = 0.1) excerpts; *t*(98) = 15, *p* < 0.0001; Effect size *d_Cohen_* = 3; 95% CI (2.19/3.81). Similarly, there was a significant difference in pulse clarity values for atonal (*M* = 0.2, *SD* = 0.1) and tonal (*M* = 0.4, *SD* = 0.1) excerpts; *t*(98) = 10, *p* < 0.0001; Effect size *d_Cohen_* = 2; 95% CI (1.32/2.68). The results represent the striven manifestation of features and demonstrate the reliability of this computational approach.

Our corpus analysis examined 50 TM excerpts from the classical period (TM, tonal music) and 50 AM excerpts (AM, atonal music). Here the main criterion was the comparability of TM and AM with regard to texture (see selection of composers and pieces in Supplementary Table 2). Since the AM pieces lack a homophonic texture and are characterized by a more independent treatment of melodic lines, we contrasted these with polyphonic pieces from the Baroque period (which corresponds to ‘TM’). Further, due to a high structural diversity within pieces, we took only 40–60 s segments into consideration for analysis. The segments were extracted so that tempo, event density and volume were kept as similar as possible within and across TM and AM segments. Moreover, excerpts were extracted to align with phrase and formal boundaries. In short, the resulting segments provide a collection of musical excerpts that are assumed to represent both TM and AM in terms of two of its core stylistic features, namely (a)tonality and the (ir)regularity of meter. The results confirmed our assumptions and demonstrate that the selected segments from TM and AM considerably differ with regards to their key and pulse clarity values (see Figure 3): Two independent-samples *t*-tests showed significant difference in key clarity values for atonal (*M* = 0.5 ± 0.1) and tonal (*M* = 0.8 ± 0.1) excerpts [*t*(98) = 15, *p* < 0.0001; *d_Cohen_* = 3; 95% CI (2.19/3.81)]. Results for pulse clarity values for atonal (*M* = 0.2 ± 0.1) and tonal (*M* = 0.4 ± 0.1) excerpts [*t*(98) = 10, *p* < 0.0001; *d_Cohen_* = 2; 95% CI (1.32/2.68)] were similarly significant.

The following part of the paper examines particular kinds of mechanisms that could contribute to a pleasurable experience with AM. However, it needs to be clarified that particularly when it comes to theories on how AM might be cognitively processed and neurally represented, a specific style of AM is being addressed. The following argumentation is based on a style of AM that does not suggest any key nor any regularity in meter (see our analyses above as well as Supplementary Table 2 for more exemplary pieces and composers). Accordingly, musical pieces from 20th/21st century art music are excluded when they do not fulfill these criteria, i.e., if they entail tonal relationships or any form metrical regularity (style example: minimal music; piece example: *Atmosphères* by György Ligeti). When comparing Western TM to AM, we refer to all music that uses the Western tonal scale and is composed due to the rules set up in the period of common practice (Hyer, 2001). However, to provide a sharp contrast the reader is encouraged, when speaking of Western TM to think of music from the common practice period. This is due to the fact that this classical or art music in contrast to more popular forms of Western TM show a higher degree of structural complexity and density, a property that it has in common with the kind of AM we focus on and thus serves as an equal comparable style.

## Accounting for Musical Pleasure of Atonal Music

Empirical or scientific approaches of the experience of pleasure with 20th/21st century art music are sparse. As mentioned in the discussion on its aesthetics, this style of music intended and continues to intend to confront the listener with unknown and novel sounds and structures.

We now examine person-related factors as well as extrinsic and intrinsic factors that have been reported to contribute to an increase in pleasure in the context of an aesthetic experience of music. Particularly, we discuss the extent to which they could account for pleasure derived from AM.

### Person-Related Factors

When considering mechanisms that bring one to engage with 20th/21st century art music, personality traits might be considered a predictive variable. With regard to music listening, individuals exhibiting high ‘openness to experience’ show a greater appreciation of complex music (Nusbaum and Silvia, 2011), are more receptive to chills and absorption (Silvia and Nusbaum, 2011) and are more likely to use music for cognitive stimulation (Rentfrow and Gosling, 2003). Moreover, devoted contemporary classical music listeners might score higher in the “Need for Cognition” (Cacioppo et al., 1996), which is allocated to the factor “openness to experience” of the NEO-PI (Mussel, 2014).

In the context of explaining creativity, Perlovsky refers to the so-called “knowledge instinct” (Perlovsky and Levine, 2012, p. 292) to describe human nature, suggesting that individuals are geared toward seeking information and discovery. It has been suggested that humans, as well as animals, inherently possess a drive for curiosity (Jepma et al., 2012; Levine, 2012) and that exploratory behavior, driven by novelty, is associated with reward in the long-term (Bevins, 2001). This trait may be more pronounced in certain individuals than in others. For instance, in creative people a relationship between “openness to experience, novelty-seeking, and preference for complexity” (Feist and Brady, 2004, p. 79) has been shown. Moreover, in behavioral genetics the trait ‘openness to experience’ (Costa and Mccrae, 1992) has been associated with modes of dopamine neurotransmission that are, in turn, associated with a “variation in reward and emotion processing” (Peciña et al., 2013, p. 878). Taken together, individuals who have a preference for AM may score high in ‘openness to experience.’ They are likely better at withstanding uncertainty and listen to AM to fulfill their enhanced need for complexity and novelty.

### Extrinsic Factors

Enjoying AM might be strongly determined by factors that can be subsumed under the effects of framing a situation (the term “frame” was coined by the sociologist Goffman, 1974). “Pre-classifications” can serve as important cues in particular in modern art (Leder et al., 2004, p. 493), so that for instance an object like the famous pissoir by Marcel Duchamp can be recognized as an art object at all. The mere fact that it is exhibited in a museum provides the relevant cue for the perception as art rather than as an utilitarian object. Mostly, such pre-classifications operate on a socio-culturally coded contextual level, like a concert hall, a gallery or a particular performative setting that takes place at a festival. Moreover, they represent the first processing stages in the context of an art experience (Leder et al., 2004; Pelowski et al., 2017).

Resulting changes in perception, evaluation and cognitive processing might appear such that individuals expect pleasurable moments, focus on particular stylistic or formal features or – which can be seen as the most important factor for the context discussed here – adopt a higher “tolerance for surprise, disgust, or ambiguity” (Pelowski et al., 2017). In the literature on music, such effects have also been defined under the term “aesthetic framing” (Juslin, 2013, p. 13) or external context (Brattico et al., 2013), mainly referring to a change in the attitude toward the music due to external factors. For instance, a concert being experienced at a famous location may lead to additional value and meaning attribution. Another important notion with regard to the aesthetic attitude is that of it being an “intentional contemplative approach to an object, which is perceived, conceptualized and evaluated as detached from its utilitarian functions” (Reybrouck and Brattico, 2015, p. 70).

Experiencing joyful moments with AM might arise from a mechanism called “cognitive mastering” (Leder et al., 2004), which in the visual domain was shown to play a major role in the enjoyment of abstract paintings. Cognitive mastering describes the capability of humans to increase their appreciation of art using cognitive constructs like information about the artwork or the artist. Cognitive constructs can also occur in the form of social constructs. In an fMRI study, evidence was found that a “cortical network typically associated with mental state attribution” (Steinbeis and Koelsch, 2009, p. 619) was more active when subjects were told that the pieces they listened to were written by a composer compared to when they were told the same pieces were written by a computer (Steinbeis and Koelsch, 2009). This suggests that cognitive constructs can potentially influence attitudes toward music and potentially lead to a higher appreciation of the artistic work.

Pleasure resulting from complex music like AM could also be understood in terms of what Brattico called “conscious enjoyment” when conceptualizing a sensory and a “conceptual hypothesis of musical pleasure” (Brattico, 2015, p. 304). There it was pointed out that the positive aesthetic experience obtainable from the *Sonata No. 1* by Pierre Boulez represents a “peculiar case of discrepancy between ‘sensory’ and ‘conscious’ enjoyment” (p. 309) and argued that although listening to an atonal piece may lead to unpleasant sensory experience linked to activations in the parahippocampal gyrus and the (right) amygdala (Brattico, 2015), individuals may still give a positive evaluation due to a particular structure they discovered or due to a repetitive pattern that created a familiarity effect. That an unpleasant sensory experience or negative emotions may be experienced differently in an aesthetic context compared to real life situations has been pointed out several times. Indeed, it has been argued that negative emotions can even reinforce the intensity of a positive aesthetic experience (Zelle, 1985; Wald-Fuhrmann, 2011; Menninghaus et al., 2017).

Processes behind aesthetic framing and cognitive mastering provide the listener with concepts and abstract information that modulate attitude and expectations *a priori* even before the music starts. We hypothesize that these processes play a crucial role in the appreciation of AM because it intrinsically requires this aesthetic attitude (Cupchik and László, 1992) being a stimulus that would not be sought out in a non-artistic environment. Framing of AM as art in combination with an aesthetic discourse that positively emphasizes complexity and novelty may be a necessary condition for this music to be enjoyed. In this regard, AM represents a compelling resource for uncovering the neural correlates of the aesthetic attitude. A behavioral first step to test the relevance of this aesthetic attitude could be to compare aesthetic judgments (e.g., liking ratings) in tonal and AM after the listeners have received information (e.g., date of composition, the piece and its historical importance, or information related to musical structure). In a between-subjects design one group would be given such information in advance whereas the other group would not receive any information before listening to the pieces. We would predict that the information has a stronger influence on liking ratings of AM than those of TM.

Finally, it has been suggested that attention in a musical experience are necessary components of the aesthetic attitude or stance (Brattico et al., 2013; Brattico E. et al., 2017; Bundgaard, 2015). Critically, AM affords an exemplary form of aesthetic musical experiences for systematic investigation since listening to AM tends to be a fully engrossing activity and is rarely done in parallel with other activities (running, doing homework and so on). This is in sharp contrast to the way with which other more popular forms of music (Schäfer and Sedlmeier, 2009; Greb et al., 2017) are engaged.

### Intrinsic Factors

#### Mere Exposure Effect

A popular explanation for listeners’ appreciation of music is that of repeated listening leading to an inevitable internalization and consequent appreciation of the musical structure. Repeated exposure has been shown to lead to an increase in liking in many domains, a finding often referred to as the *mere exposure effect* (Zajonc, 1968). Increasing familiarity with a stimulus can effectively lead to liking even in the case of dissonant music (Omigie et al., 2017). Since AM entails a high degree of novelty and is accordingly difficult to remember, repeated listening may even more than in the case of TM, play a crucial role in appreciation and preference. Specifically, listeners might learn a given piece of music to the extent that they can form veridical expectations (i.e., piece-specific expectations; Snyder, 2000) at certain points in the music, while other less learned moments may still induce a (pleasurable) prediction error (PE). Moreover, general familiarity with AM as a musical style – resulting from repeated listening of single pieces – contributes to an increase in liking since listeners begin to form schematic expectations (Snyder, 2000; Huron, 2006).

Learning mechanisms would facilitate stimulus perception and processing, allowing appreciation to arise from ‘processing fluency,’ the notion that enjoyment of art can result from smooth processing (Reber et al., 2004). One study from the computational modeling domain supports the notion that learning mechanisms have considerable effects on the processing of serial music: a computational model that had been trained on a corpus of artificially composed serial music pieces was able to better predict pieces of Arnold Schoenberg and Anton Webern (Dean and Pearce, 2016) than a corpus which was trained on tonal pieces. This suggests that an exposure to a serially composed structure could lead to a more predictable listening experience. This predictability in turn allows a better fluency in processing which has been hypothesized to be linked with a pleasurable response. The more fluent an artwork can be perceived, the more pleasure the perceiver obtains from the object (Reber et al., 2004). Since processing fluency is positively influenced by variables such as symmetry, repetition, figural goodness and even by priming procedures, it is likely a critical factor for perceptually challenging stimuli like AM. Similarly, Daniel Berlyne’s theory of optimal arousal (Berlyne, 1971) states that the pleasantness of a stimulus is influenced by properties of the stimulus that he called “collative or structural properties” (Berlyne, 1971, p. 81). These properties influence the stimulus’ arousal potential and accordingly its hedonic value. The optimal arousal represents the most pleasurable state and occurs when the stimulus has reached a medium level between simplicity and complexity, as well as between familiarity and novelty (Berlyne, 1970). Atonal music might result in a high arousal state due to its high complexity and high novelty (Berlyne, 1954) at least partially explaining why most music listener have no preference for this kind of music. However, by virtue of repeated exposure, this complexity would decrease and familiarity and predictability would increase. This could lead to a medium arousal level and accordingly result in an increase in hedonic value.

In contrast to aesthetic framing and cognitive mastering, mechanisms like mere exposure result in increased processing fluency and can be said to unfold their effects during the listening process. This listening process will be the focus of the following chapters. There, the cognitive and neuronal processes involved in processing stimuli with a high degree of uncertainty will be described in greater detail.

#### Uncertainty as a Source of Pleasure

Recent approaches to musical pleasure have emphasized the role of PE in the musical aesthetic experience. This is perhaps because the majority of neuroscience research on musical pleasure to date has been carried out with Western major-minor music, which has a clear tonal structure that can be “played with” or can be broken (Vuust and Kringelbach, 2010; Koelsch, 2012; Rohrmeier and Koelsch, 2012). In the following, we will first examine the relation between prediction and musical pleasure, demonstrate which role the PE possibly plays in listening to AM and finally show which neural mechanism might drive the encounter as well as enable pleasurable experience within uncertain environments.

##### Prediction and pleasure in music listening

In the context of TM, researchers have been able to show that peak moments of music-induced pleasure (indicated by participants as chills-inducing) activate the reward and limbic circuits of the human brain regulated by dopamine neurotransmission, and specifically the striatum (Blood and Zatorre, 2001; Salimpoor et al., 2011). In more detail, this research has revealed that the anticipation of musical peak experiences has specific neural correlates in the caudate nucleus (dorsal striatum), whereas the peak experience itself is associated with activity in the nucleus accumbens (ventral striatum, Salimpoor et al., 2011). Salimpoor et al. (2011) speculated that this shift in activity from the dorsal to ventral striatum, accompanying the buildup and experience of peak musical experiences, reflects expectancy and consummatory processes that are central in music perception (Meyer, 1956; Huron, 2006).

While the studies above were seminal in linking, for the first time, dopamine-regulated activity of the nucleus accumbens to pleasurable music listening experiences, they were also compelling in their interpretation of the activity of the reward system in the context of reward PE and anticipatory processes (Meyer, 1956; Huron, 2006; Vuust et al., 2009). This hypothesis about dopamine release is now prevalent in accounts of music-induced emotions (Juslin and Västfjäll, 2008; Gebauer et al., 2012; Rohrmeier and Koelsch, 2012; Salimpoor et al., 2015) and derives from the observation in animal models that an unexpected reward generate a dopamine burst more than a fully expected rewarding outcome (Schultz et al., 1997; Schultz, 1998).

A related theory, not directly linked to pleasure (Ross and Hansen, 2016), claims that the brain is a Bayesian machine, anticipating and inferring upcoming events based on the statistics of previous sensory input (Clark, 2013). This predominant theory of brain function states that the brain continuously predicts what comes next, producing models (or priors) of the environment that are updated after errors occur. This theory called predictive coding is used to account for cortical responses to unexpected or oddball events. Specifically, it has been used to explain the mismatch negativity (MMN), an early brain response to regularity violations that is held to index the formation and continuous updating of predictive models of the auditory environment (Näätänen, 2003; Vuust and Frith, 2008; Garrido et al., 2009; Bendixen et al., 2012; Rohrmeier and Koelsch, 2012; Lieder et al., 2013).

The theory of predictive coding states that, as a “prediction machine,” the brain’s main function is to track and to predict changes in the environment in order to ensure optimal adaptation (Friston, 2009; Clark, 2013). A key assumption of the theory is that the brain constantly predicts incoming sensory input based on generative cognitive models on higher levels. These top–down predictions continuously encounter bottom-up sensory input and in the event that the model has generated an incorrect prediction, a PE signal is used to update the model (Kopell et al., 2000; Fontolan et al., 2014).

##### Precision-weighting of PE in music

When considering music listening in predictive coding terms, it is important to note that predictive models are highly dependent on the probabilistic distributions of the incoming sensory input. Recent theories of predictive coding emphasize that the “neural estimations of the reliability of those predictions” are just as important as predictions themselves (Clark, 2016, p. 9). In other words, the extent to which we rely on our own predictions is critical. The estimation of the degree to which we are certain or uncertain in our predictions and the modulation of gain in neural responses relative to this certainty is referred to as the precision-weighting of PE (Friston, 2009).

The notion of the importance of the precision-weighting of PE is supported by findings that the brain effectively adapts to the degree of predictability of the stimulus and that statistical properties of the stimulus influence the precision of prediction (Garrido et al., 2013; Sohoglu and Chait, 2016; Heilbron and Chait, 2017). Specifically, Hsu et al. (2015) were able to show this clearly in a study in which they recorded brain responses to target notes in three conditions: the first two in the context of low uncertainty sequences (whereby the target note was either predicted or mispredicted) and the last in the context of a high uncertainty sequence (where the target note was unpredicted). The authors revealed that the amplitude of the N1 response differed across conditions with the largest amplitude observable for the mispredicted note, the medium response for the predicted and the most attenuated response for unpredicted stimuli. The finding that PE activity is related to the uncertainty of the ongoing context (Hsu et al., 2015) is highly relevant for understanding how AM could potentially be processed.

From the perspective of evolutionary biology, uncertainty estimation is a vital necessity. It has been suggested that uncertainty and uncertainty estimation is essential for living organisms (Fiorillo et al., 2003; Bach and Dolan, 2012). It suggested that as they often encounter situations of uncertain reward outcomes in everyday life, it is important that organisms are able to track reward probabilities in order to maximize outcomes (Tobler et al., 2005). The theory holds that uncertainty motivates agents to learn and to explore new environments, ultimately to reach long term goals (Bach and Dolan, 2012). With regard to what form a mechanism for reward from uncertainty might take, animal research has shown dopaminergic activity to track highly uncertain situations. Fiorillo et al. (2003) showed that midbrain dopamine neurons show two distinct response types in the context of probabilistic reward receipt; one, a brief and phasic activation tracking increasing reward probability, and the other a slower and more sustained response tracking increasing reward uncertainty: “The sustained, uncertainty-induced increase in dopamine could act to reinforce risk-taking behavior and its consequent reward information” (Fiorillo et al., 2003, p. 4). These risk-taking behaviors are advantageous for learning and exploration and are from an evolutionary perspective distinguished from exploitation, which “is the time spent using behaviors with known reward values” (Kringelbach and Berridge, 2009, p. 205).

However, it is unlikely that listeners of AM encounter an entirely unpredictable and uncertain auditory environment as used in the “unpredicted” condition of the study of Hsu et al. (2015), where random tone sequences were used. Since music consists of more parameters than tonal relationships, predictions during music listen may rely on other more global features like timbre, dynamics, musical gestures or groupings based on pitch proximity or rhythm (Brattico P. et al., 2017). These low- to mid-level features may often play a key role in predictive processes serving as the anchoring points of predictive models for AM. In fact, these features may create a hierarchy of their own and could represent what Bharucha and others call “event hierarchies” (Bharucha, 1984; Deutsch, 1984) which represents a counterpart of the tonal hierarchies within a piece of music (Krumhansl and Cuddy, 2010). The extent to which predictive models based on event hierarchies differ from those of tonal hierarchies is a highly interesting issue that needs theoretical and empirical investigation, as argued in a recent work (Brattico P. et al., 2017). Nevertheless, compared to music with clear tonal relationships, the degree of predictive uncertainty in the context of these low- to mid-level features might still be very high (Imberty, 1990) and the lack of tonal relationships as well as the missing metrical regularity strongly complicates prediction processes. As a consequence, AM from the 20th and 21st century might be classified as a music that provides a listener with a rather weak predictive model stemming from the absence of tonal and metrical regularity and resulting in a fairly high amount of uncertainty.

##### Prediction mechanisms in atonal music

In Western TM, tonal hierarchies afford a strong predictive model which can result in certain events being perceived as highly unexpected. Moreover, points of high information content (e.g., unexpected harmonic changes (Sloboda, 1991) in musical pieces have been shown to generate higher physiological arousal states (Egermann et al., 2013) associated with highly pleasurable chills and shivers (Grewe et al., 2009). With regard to highly irregular AM, naïve listeners might initially experience a high number of PEs, as a result of continuing to apply tonal schemas and expectations to the AM (Ockelford and Sergeant, 2012). However, potentially even shortly after introduction to AM, experienced TM listeners’ predictive neural processes may adapt to the tonal and metrical uncertainty of AM, since the brain is known to internalize the statistics of the environment relatively quickly (Hsu et al., 2015; Barascud et al., 2016; Pearce, 2018). This might lead to a weaker predictive model and accordingly to attenuated predictions as neural processes successfully adapt to the irregularity. We assume that particularly in the case of strongly AM which additionally lacks a metrical structure, a weak predictive model and consequent attenuated predictions might be reflected in neural activations level.

However, in the case of PEs that are linked to pleasurable chills, which properties must an uncertain stimulus fulfill to produce pleasurable PEs? Presumably, once adapted to the music, listeners might expect irregularity or “the unexpected” (Huron, 2006). The question arises how pleasurable PEs can occur when no or only weak predictions are created. Paradoxically, the most unlikely event or series of events to happen in a highly irregular environment is increased regularity. Regularity offers the opportunity to improve the model and might therefore be evaluated as positive. Entry of a regular pattern would stick out perceptually and give the listener the opportunity to generate predictions. This may be expected to result in a rewarding subjective state. Previous work shows that a shift from a random to a regular structure results in increased sustained neural activity (Barascud et al., 2016) and may reflect the improved momentary predictive model the perceiver has.

Taken together, we hypothesize that in AM where only weak predictive models can be maintained, the occasional occurrence of regularities (e.g., a tone that keeps on repeating throughout a piece; a specific timbre that occurs from time to time) may be linked to pleasurable moments, since they allow generation of stronger predictions and since the individual is given the opportunity to update their predictive model and increase prediction certainty. To test the notion of the relevance of correct predictions for pleasure in high uncertainty contexts, one could generate strongly atonal stimuli with varying degrees of recognizable patterns. The more recognizable the pattern, the more the piece may be liked. Moreover, when manipulating pieces in such a way that different patterns are built on separate low-to-mid level features (rhythmical, dynamical, pitch grouping level) constituting different sorts of event hierarchies (Bharucha, 1984; Deutsch, 1984), one could investigate the strengths of different predictive models belonging to different event hierarchies. How this is shaped by long-term exposure might be revealed by investigating contemporary music aficionados, as well as by musicians who are experts in the repertoire of 20th and 21st century art music.

### Synthesis: Appreciation of Atonal Music

In the previous sections, we described a number of different factors that may contribute to a pleasurable experience of AM. Personality traits like “openness to experience” or a higher score in “need for cognition” may drive the motivation for engagement with art in general and 20th/21st century art music in particular. Atonal music may therefore be linked to (auditory) exploratory behavior. Having claimed that pattern detection is the central mechanism that affords pleasurable moments with AM, working memory capacity may also be a strong predictor of deriving enjoyment from this music. Furthermore, extrinsic factors like framing and cognitive mastering may function as cues and serve as anchors and reference points throughout the listening experience. They determine attitudes in advance, might trigger “perceptual curiosity” (Jepma et al., 2012) as well as “interest” (Silvia, 2008) and may promote motivation and willingness to engage with the music. They may also enable the listener to create some expectations and predictions that in turn enhances the listeners predictive model as they use PEs to refine it. Complementarily, our particular focus with regard to intrinsic factors was to demonstrate that AM does not generate the strong predictive models (on the basis of key and rhythm) that TM does and that the listener encounters a state of rather high uncertainty. Since uncertainty estimation is an evolutionary necessity for explorative behavior (Tobler et al., 2005; Bach and Dolan, 2012) we argue that processing and decoding uncertainty in (atonal) music primarily represents an incentive to further engage with this stimulus. Whether one can speak of pleasure arising from this process remains an open question. Here, we argue with greater conviction that pleasure from AM may arise from the rare recognition of regular structures and underlying patterns. Such moments of increased regularity offer the opportunity to turn a weak into a strong predictive model.

In closing, addressing the case of AM clearly illustrates that a pleasurable experience with music can be elicited in different ways: while TM which offers a strong predictive model may require wrong predictions to lead to some pleasurable states, for AM which offers the listener only a weak predictive model, correct predictions may be more likely than wrong predictions to lead to enjoyment. Thus, we emphasize here the importance of the strength of a predictive model in predetermining the mechanisms by which pleasure may be induced during music listening.

## Future Prospects

We would like to motivate research into art music from the 20th/21st century for several reasons. Firstly, we suggest thata comprehensive understanding of the aesthetic experience can arguably only be achieved after acknowledging and thus investigating the diversity of artistic phenomena. As art music from the 20th and 21st century follows a very different aesthetic paradigm to Western TM, but is rooted in the same cultural context, this expansion can be expected to reveal novel insights into the aesthetic experience of music in general. Moreover, as a result of the specific properties of AM examined in this paper, we suggest that the pre-classification and aesthetic framing of AM as art facilitates the appreciation of what may otherwise be considered an unattractive property and argue that this paradox makes this music a unique musical style with which to reveal key underlying mechanisms of musical aesthetic experience.

Second, the process of listening to AM represents a compelling approach with which to investigate states of uncertainty, where individuals are required to not only withstand such uncertain states but also to remain motivated to seek out coherence and underlying structural patterns in order to improve their predictive models. Atonal music could serve as a tool to investigate learning and pattern recognition mechanisms and could moreover help to unravel a variety of factors that contribute to the appreciation of such uncertain environments.

Finally, in addressing such an extreme form of music, it becomes clear that the pleasure derived from music may come from multiple facets resulting in an equally large number of forms of positive experiences. In order to investigate such different qualities of musical pleasure, we would like to suggest the importance of taking into account the precision of predictions a musical piece or musical style allows. Thus, we suggest that the strength of a predictive model may not only be used to predict the degree and quality of pleasure induced by music over time but also the distinct mental states that different aesthetic experiences may be characterized by.

## Author Contributions

IM conceived and developed the idea for this manuscript and prepared the first draft. DO added substantial content and contributed with drawing up the manuscript. EB contributed to the computational feature analysis. EB and MW-F provided revisions for important intellectual content. All authors gave final approval of the manuscript to be published and have agreed to be held accountable for the accuracy and integrity of this manuscript.

## Conflict of Interest Statement

The authors declare that the research was conducted in the absence of any commercial or financial relationships that could be construed as a potential conflict of interest.

## Footnotes

1 The types of music that are in the center of this article are generally referred to with various terms – in scholarship as well as in music criticism and the public discourse about music: Modernism, Twelve-Tone Music, Serialism, Neue Musik, avant-garde music, atonal music, or contemporary music in the Western tradition (Taruskin, 2005). Broadly speaking, they comprise Western art music of the 20th and early 21st century. Although atonality does not apply to all styles of 20th/21st century music, it can be seen as one defining element of the avant-garde strand of modernist music that evolved in Vienna around 1911 and has influenced avant-garde music before 1945 and continues to influence avant-garde music up until today. In this article we therefore mainly speak of three types of music: (1) 20th/21st century art music (Clarke, 2017) is being used to refer to the entire phenomenon of 20th and 21st Western art music. In contrast, (2) the term ‘atonal music’ occurs in a more specific context, particularly in chapter 4 and 5, where the property of atonality (music that lacks a tonal centre; Forte, 1977; Forte, 1998) is a crucial aspect in our argumentation. Finally, (3) the term ‘contemporary classical music’ (in the Western tradition) is being used, when there is a clear emphasis on recent artistic practice.

2 Not only do musical styles and strands not possess a clear definition as addressed in footnote 1, but equally the terms *tonality* and *atonality* have no sharp definition. For the purpose of this paper, however, the following explanation tries to clarify our usage of these terms: When we address tonal music we mainly refer to musical styles of the Western culture from the 17th to the early 20th century (and in some musical genres, specifically genres of popular music, even until today). Due to the usage of a tonal scale, most of the music has a tonal centre (the tonic) and thus hierarchical relations between pitches and chords. This can be seen as one of the rare but essential characteristics that is linked to the concept of tonality (Dahlhaus, 1967; Hyer, 2001). Complementarily, when writing about atonal music, we are referring to those kinds of 20th/21st century art music that are characterized by the lack of a tonal scale, tonal centre and a regular meter.

3 See reviews in newspapers and journals that report about contemporary classical music. German-speaking countries for instance: *neue musik zeitung*, *Neue Zeitschrift für Musik*, *Positionen*, *Musiktexte – Zeitschrift für Neue Musik.* English-speaking area: *Tempo*, *Musical Times*.

4 However, it is worth noting that the AM pieces, particularly Figures 1E,F, display some kind of musical gestures. Even though the groups of notes are not made up by tonal hierarchies, there is still a formation happening that could be called a musical gesture (Godøy and Leman, 2010).
