# Supplementary material for: Atonal Music: Can Uncertainty Lead to Pleasure?
Source: Front Neurosci. 2019 Jan 8;12:979. doi: 10.3389/fnins.2018.00979 (PMC6331456; doi:10.3389/fnins.2018.00979)
Supplement: Supplementary file 1 [file Data_Sheet_1.pdf]

Supplementary Material

Modern Music: Can uncertainty lead to pleasure?

Iris Mencke, Diana Omigie, Elvira Brattico, Melanie Wald-Fuhrmann

*** Correspondence:** Iris Mencke: iris.mencke@ae.mpg.de

Supplementary Tables

Supplementary Table 1: List of audio recordings used for MIR toolbox single piece analysis.

Composer	Work	Performer	Title of CD	Label
J. S. Bach	Goldberg Variations, Variatio 12	Alexandre Tharaud	Bach: Goldberg Variations	Erato
W. A. Mozart	Piano Sonata No. 8, K. 310	Fazil Say	Mozart: Complete Piano Sonatas	Warner
F. Chopin	Waltz, A-Minor	Makiko Takedo-Herms	Die 15 Walzer von Frédéric Chopin	Streetlife-Music
K. Stockhausen	Piano Piece No. VIII	Steffen Schleiermacher	Bass, Clarinet & Piano	MDG
W. Rihm	Piano Piece No. 5	Eduard Steuermann	Darmstadt Aural documents	Neos Musik
J. Widmann	Fleurs du Mal	Jan Philip Schulze	Piano Works	Neos Musik

Supplementary Table 2: List of pieces and excerpts used for the MIR toolbox corpus analysis.

ATONAL MUSIC

Composer	Title	Starting time	End time	Total length
Arnold Schönberg (1874 - 1951)	Drei Klavierstücke Op. 11, I	00:00	00:41	41
Arnold Schönberg	Drei Klavierstücke Op. 11, III	00:19	01:02	43
Arnold Schönberg	Fünf Klavierstücke Op. 23, I	00:00	00:42	42
Arnold Schönberg	Fünf Klavierstücke Op. 23, II	00:00	00:45	45
Arnold Schönberg	Fünf Klavierstücke Op. 23, III	00:01	00:47	46
Arnold Schönberg	Fünf Klavierstücke Op. 23, IV	01:44	02:25	41
Arnold Schönberg	Fünf Klavierstücke Op. 23, V	00:00	00:44	44
Arnold Schönberg	Klavierstück Op. 33a	00:00	00:43	43
Arnold Schönberg	Klavierstück Op. 33b	01:27	02:14	47
Anton Webern (1883 - 1945)	Variationen für Klavier Op. 27, III	00:30	01:16	46
Luigi Dallapiccola (1904 - 1975)	Quaderna musicale di Annalibera, III	00:00	00:44	44
Luigi Dallapiccola	Quaderna musicale di Annalibera, VII	00:00	00:45	45
Ernst Krenek (1900 - 1991)	20 Miniatures Op. 139, IX	00:03	00:50	47
Oliver Messiaen (1908 - 1992)	Mode de valeurs et d'intensités	00:00	00:43	43
Karel Goeyvaerts (1923 -1993)	Sonata No. 1 Op. 1	03:27	04:13	46
Luciano Berio (1925 - 2003)	5 Variazioni	00:00	00:40	40
Luciano Berio	Sequenza	08:38	09:23	45
Luciano Berio	Six encorespour piano, Brin	00:09	00:54	45

Luciano Berio	Six encorespour piano, Leaf	00:00	00:44	44
Pierre Boulez (1925 - 2016)	Klaviersonate Nr. 2, II	00:00	00:49	49
Pierre Boulez	Klaviersonate Nr. 2, III	00:00	00:45	45
Pierre Boulez	Klaviersonate Nr. 2, IV	09:31	10:14	43
Pierre Boulez	Notations, III	00:00	00:43	43
Morton Feldman (1926 - 1987)	Last Pieces, II	00:00	00:46	46
Morton Feldman	Last Pieces, IV	00:00	00:44	44
Morton Feldman	Piano piece to Philip Guston	00:00	00:44	44
Hans Werner Henze (1926 - 2012)	Lucy Escott Variations	02:11	03:00	49
Hans Werner Henze	Cherubino II	00:07	00:52	45
Hans Werner Henze	Sonata per pianoforte I	00:00	00:46	46
Hans Werner Henze	Sonata per pianoforte II	01:39	02:20	41
Hans Werner Henze	Sonata per pianoforte III	00:00	00:41	41
Karlheinz Stockhausen (1928 - 2007)	Tierkreis	01:10	01:50	40
Karlheinz Stockhausen	Klavierstück V	02:10	02:52	42
Karlheinz Stockhausen	Klavierstück X	01:01	01:42	41
Karlheinz Stockhausen	Klavierstück I	00:00	00:40	40
Karlheinz Stockhausen	Klavierstück II	00:00	00:44	44
Karlheinz Stockhausen	Klavierstück IV	00:00	00:46	46
Karlheinz Stockhausen	Klavierstück V	00:26	01:09	43
Karlheinz Stockhausen	Klavierstück VIII	00:39	01:22	43
Brian Ferneyhough (*1943)	Lemma-Icon-Epigram	00:00	00:40	40
Brian Ferneyhough	Quirl	02:05	02:48	43
Brian Ferneyhough	Invention	00:00	00:46	46
Brian Ferneyhough	Epigram VI	00:30	01:13	43
Salvatore Sciarrino (*1947)	Klaviersonate III	00:41	01:21	40
Beat Furrer (*1954)	Phasma	07:58	08:46	48
Jörg Widmann (*1973)	Fleurs du mal: Piano Sonata after Baudelaire	02:21	03:09	48
Jörg Widmann	Elf Humoresken: 1. Kinderlied	00:00	00:42	42
Rodion K. Schtschedrin (*1932)	24 Präludien und Fugen, XIII	00:13	00:58	45
Rodion K. Schtschedrin	Polyphonisches Spielheft, II	00:00	00:45	45
Rodion K. Schtschedrin	Polyphonisches Spielheft, VI	01:12	01:53	41

TONAL MUSIC

Composer	Title	Starting time	End time	Total length
Johann Sebastian Bach (1685 - 1750)	Fuge in C BWV 846	00:50	01:31	41
Johann Sebastian Bach	Fuge in c BWV 847	00:00	00:46	46
Johann Sebastian Bach	Fuge in Cis BWV 848	00:00	00:43	43
Johann Sebastian Bach	Fuge in cis BWV 849	00:13	00:56	43
Johann Sebastian Bach	Fuge in d BWV 851	00:15	01:01	46
Johann Sebastian Bach	Fuge in dis BWV 853	00:08	00:55	47
Johann Sebastian Bach	Präludium in E BWV 854	00:32	01:17	45
Johann Sebastian Bach	Fuge in F BWV 856	00:00	00:44	44
Johann Sebastian Bach	Präludium in f BWV 857	00:00	00:41	41
Johann Sebastian Bach	Fuge in f BWV 857	00:12	00:59	47
Johann Sebastian Bach	Fuge in Fis BWV 858	00:04	00:53	49
Johann Sebastian Bach	Fuge in G BWV 860	00:00	00:45	45
Johann Sebastian Bach	Fuge in As BWV 862	00:05	00:53	48
Johann Sebastian Bach	Präludium in gis BWV 863	00:40	01:27	47
Johann Sebastian Bach	Fuge in gis BWV 863	00:00	00:43	43
Johann Sebastian Bach	Präludium in A BWV 864	00:00	00:40	40
Johann Sebastian Bach	Fuge in A BWV 864	00:00	00:46	46

Johann Sebastian Bach	Fuge in B BWV 866	00:53	01:35	42
Johann Sebastian Bach	Präludium in H BWV 868	00:07	00:53	46
Johann Sebastian Bach	Fuge in H BWV 868	00:24	01:07	43
Johann Sebastian Bach	Fuge in h BWV 869	00:00	00:49	49
Johann Sebastian Bach	Präludium in c BWV 871	01:04	01:46	42
Johann Sebastian Bach	Fuge in c BWV 871	00:56	01:38	42
Johann Sebastian Bach	Fuge in Cis BWV 872	00:00	00:43	43
Johann Sebastian Bach	Präludium in cis BWV 873	00:02	00:51	49
Johann Sebastian Bach	Fuge in D BWV 874	00:44	01:30	46
Johann Sebastian Bach	Fuge in d BWV 875	00:17	01:00	43
Johann Sebastian Bach	Fuge in dis BWV 877	00:00	00:44	44
Johann Sebastian Bach	Präludium in E BWV 878	00:10	00:55	45
Johann Sebastian Bach	Fuge in E BWV 878	00:15	01:03	48
Johann Sebastian Bach	Präludium in Fis BWV 882	00:00	00:46	46
Johann Sebastian Bach	Fuge in Fis BWV 882	00:00	00:48	48
Johann Sebastian Bach	Fuge in As BWV 886	00:00	00:42	42
Johann Sebastian Bach	Fuge in gis BWV 887	02:12	02:52	40
Johann Sebastian Bach	Präludium in A BWV 888	00:00	00:42	42
Johann Sebastian Bach	Päludium in a BWV 889	00:36	01:21	45
Johann Sebastian Bach	Fuge in B BWV 890	00:00	00:41	41
Johann Sebastian Bach	Präludium in b BWV 891	00:00	00:42	42
Johann Sebastian Bach	Fuge in b BWV 891	01:21	02:03	42
Johann Sebastian Bach	Fuge in H BWV 892	00:00	00:49	49
Anton Reicha (1770 -1836)	Fuge in d op. 36, Nr. 10	00:00	00:43	43
Anton Reicha	Fuge in C op. 36, Nr. 21	00:00	00:42	42
Anton Reicha	Fuge in d op. 36, Nr. 29	00:00	00:42	42
Franz Schubert (1797 - 1828)	Fuge in e, D 952	01:44	02:29	45
Robert Schumann (1810 -1856)	Fuge in d Op. 72, Nr. 1	00:04	00:51	47
César Franck (1822 - 1890)	Fuge in h op. 18, Nr 2	00:39	01:20	41
Dmitri D. Schostakowitsch (1906-1975)	Fuge in C op. 87, Nr. 1	00:49	01:32	43
Dmitri D. Schostakowitsch	Fuge in e op. 87, Nr. 4	00:34	01:19	45
Dmitri D. Schostakowitsch	Fuge in h op. 87, Nr. 6	00:08	00:55	47
Dmitri D. Schostakowitsch	Fuge in cis op. 87, Nr. 10	00:12	00:59	47

Supplementary Table 2: List of pieces and excerpts used for the MIR toolbox corpus analysis.

This table lists the musical material that was used for the corpus analysis of AM and TM piece excerpts. Next to composer and piece title both the starting and end time (min | sec) as well as the total length of the excerpts (in seconds) is specified.
